# Radiotherapy in Some Malignant Conditions of the Mouth

**Published:** 1904-12

**Authors:** Milton Franklin

**Affiliations:** New York


### International Dental Journal.

Vol. XXV. December, 1904. No. 12.

### Original Communications.[1]

### RADIOTHERAPY IN SOME MALIGNANT CONDITIONS OF THE MOUTH.[2]

BY MILTON FRANKLIN, M.D., NEW YORK.

The now almost universally accepted theory of the action of the X-ray upon the human organism supposes that in all tissue exposed there is effected a high degree of stimulation, which, when continued for a time disproportionate to the resistance of the affected tissue, assumes the proportions of an inflammation, of gradually increasing severity, finally ending in sloughing and necrosis.

Whether the tissue be normal or pathological, whether the result be beneficial or destructive, the action of the ray is the same, and can be said to differ only in degree. Assertions that there exist a distinct curative and burning action are erroneous.

A consideration of the phenomena which occur in an area that has been exposed to the action of the rays will elicit the fact that they strikingly resemble in character and sequence those of typical

---

[1] The editor and publishers are not responsible for the views of authors of papers published in this department, nor for any claim to novelty, or otherwise, that may be made by them. No papers will be received for this department that have appeared in any other journal published in the country.

[2] Read before The New York Institute of Stomatology, June 3, 1904.

classic inflammation. There is the same congestive hyperæmia, followed by the slowing of the blood-current, diapedesis, and vascular exudation, with their accompanying alteration in the vascular walls. There is the increased flow of leucocytes to the part and the infiltration of the tissues by them. These changes are followed in turn by the other well-known changes of inflammation, and may extend through the stages of fibrinous exudation or even to the formation of pus followed by necrosis with sloughing. The causes of inflammation are various, such as traumatism, the action of strong chemicals, heat, electricity, and the X-ray. Each of the different causative agents gives rise to a somewhat different train of phenomena, and while the course of inflammation is in the main the same in each, the different causes may be generally recognized from the resulting inflammation. So also in the case of the X-ray, there are certain peculiarities which render the character of the inflammation slightly different from the rest.

This difference, as far as can be deduced from facts thus far known, consists in that the action commences generally only after a varying period of latent inactivity, that the effect seems entirely due to action upon the nerve terminals, and that the resolution of the scar so closely resembles that of healthy tissue that it is unlike any class of scar heretofore observed, both in absence of tissue loss and of tendency to hypertrophy.

Besides this action in causing inflammation through direct stimulation, there is the almost paradoxical coincident inhibitory effect upon lower forms of cell life. This action has been so certainly established that I shall not attempt to detail the experiments which have led to a knowledge of the fact.

In the main these effects are common to the X-ray, the ultraviolet ray as applied by Finsen, and to the emanations of radium. In the case of the X-ray the destructive action is dominant; in the case of the ultra-violet ray there seems at the present time to be strong reason to suppose that the germicidal factor plays no inconsiderable part, while the ability to cause the growth of almost normal new tissue in the area formerly occupied by the diseased tissue is quite remarkable, excelling even that of the X-ray. In the case of radium there has been so little evidence up to the present time that is likely to prove of any therapeutic value, that I shall not consume the time or space necessary to discuss it. At the same time it may be well to point out that on account of the small size of the tubes, combined with their independent activity, apart from any connection with an outside source of electricity, they might prove of interest in conditions of the mouth if they are ultimately found to possess any value in medical science.

In considering which method to use when dealing with a malignant condition, much judgment should be exercised. The disadvantages and limitations of operative methods should be carefully compared with those of the X-ray, and the method chosen which, in the case under consideration, offers the greatest number of advantages and the fewest disadvantages.

In the treatment of diseased tissues with the X-ray, it should be remembered that all tissues are affected in the same way, the action being the same upon healthy as upon morbid tissue. The different effects are attributable, in the main, to the lower vitality of the latter. The changes that are gone through follow each other with comparatively greater rapidity in the less resisting tissue, while the normal tissue is but mildly stimulated. Thus the stage of sloughing and cell proliferation may be reached in the tumor, while in the adjacent healthy tissue, equally exposed, there may be only mild irritation, or, at the most, burn of the first degree. At the same time the retarding action upon lower forms of cell life should be taken into consideration as probably inhibiting the growth of the neoplasm.

The advantages of surgical measures over the X-ray may be briefly summed up as follows:

1. Celerity. The whole diseased tissue as well as the threatened tissue may be removed at once, while in the case of the ray, weeks, months, and in some few cases a year, or even more, has been necessary.

2. Conservation of the patient's strength. By removing from the body, at one operation, the whole malignant growth, a great drain upon the patient is avoided, together with the constant danger of metastasis.

3. The removal of the growth from the body by an outside route instead of causing it to be eliminated by the body organs, with the consequent accompanying autointoxication from the presence of the degenerated noxious tissue products. The strain upon the eliminating organs and the danger of metastasis, which it is now generally accepted is increased by the action of the ray, is avoided.

The considerations in favor of the ray method are briefly as follows:

1. Avoidance of surgical shock and anæsthetic complications.

2. Better ability to differentiate, and assurance that only the diseased tissue will be destroyed.

3. Better appearance of the resulting scar, which in the majority of cases so far resembles the normal tissue as to almost entirely escape detection.

Considerations of locality will in a measure determine which of the two methods is to be preferred in some cases, it being remembered that the ray cannot be focussed, and that the tissue surrounding the area to be treated should be protected as far as possible from the action of the ray.

In deep-seated conditions the ray as a curative agent cannot be too strongly condemned. The necessity of traversing healthy tissue by the inflammatory rays subjects them to the great danger of burn, with the accompanying inflammatory changes. Besides this the delicate nature of the lining membranes of the bodily viscera renders them liable to so great irritation as to endanger life. Besides this there is no authentic case of cure of deep cancer at present extant.

The determination of which method is to be used in any instance should be based upon a consideration of the features of the case in connection with the characters of the methods available.

In general terms cases in which the X-ray is to be recommended are those in which (1) the condition is essentially superficial; (2) where the loss of any great amount of tissue would be serious; (3) where there is little likelihood of other organs being involved through metastasis; (4) where cosmetic considerations outweigh all others; (5) where time is a negligible quantity and does not add danger; and (6) where the case is for any reason inoperable. In general all other cases are preferably treated by operative methods.

I make mention here of only those conditions of the mouth in which, in my opinion, there is nothing to be gained by employing the radiotherapeutic in preference to the operative method. There are numerous other cases where either some difference of opinion exists, or the ray is generally accepted as inapplicable.

Epithelioma of the mouth, when situated on the edge of the lip, is frequently better treated with the ray than by other methods.

Here the loss of tissue should if possible be avoided. The condition is among the most amenable to treatment by the ray, and the superficial character renders it the more so. In cases where there is much involvement of the submaxillary glands, while it is the experience of some practitioners that the size of the glands decreases simultaneously with that of the lesion, it is better practice to extirpate the whole of the threatened and diseased tissue and then apply the ray for the purpose of preventing recurrence. Where the epithelioma involves the tongue, it is preferable to use the rays where conditions do not specifically contraindicate. Here any loss of tissue is followed by grave consequences, and the results in cases that have been treated by the rays have been such as to render the method of unquestionable value. In cancer of the larynx or pharynx, notwithstanding some reputed cures, it is certain that there is little to be gained by using the ray. Because of the impossibility of applying the rays direct, but only through the tissues of the neck, it is doubtful if any benefit can be obtained without seriously impairing the tissues traversed. The ray may be used after the operation to lessen the likelihood of recurrence. This is in no way inconsistent with anything that has been said regarding the danger of raying deep-lying structures, as it is necessary to apply the rays only a few times to obtain reasonable assurance.

In noma the results of raying have, on the whole, been of negative character. Here the utmost despatch is needed in ridding the mouth of the offending mass, as any delay is apt to result in irreparable loss of tissue or even of life.

Catarrhal and croupous stomatitis, though not malignant diseases, are worthy of mention here because of the striking manner in which they respond to the ultra-violet treatment of Finsen. In the former condition the degeneration of the epithelium and the formation of pus cease after a few treatments, and the substitution of a new membrane follows speedily. In the croupous condition the false membrane melts away under the action of the light, and is followed by no perceptible scar. Where there has been tendency to necrosis, there appears a cicatrix after the cessation of the morbid process, which closely resembles the normal tissues.

Tubercular stomatitis is readily influenced by the ultra-violet ray, and as there is rarely any communication of the process to other parts of the body, there is little danger in a prolonged treatment. It is therefore preferable to proceed by phototherapeutic methods instead of resorting to operation. The X-ray also exercises a marked benefit upon these cases, but on account of the danger of burning the face, the ultra-violet rays are to be preferred.

Papillomata are best treated by the X-ray when they are of small extent, and by operation when large. The ray is recommended, as the results are obtained in small tumors before the lining membrane of the mouth is affected, and in these cases this method is simpler than operation.

Angiomata respond very readily to the action of the X-ray, the internal walls of the vessels undergoing a change resulting in early occlusion, followed by atrophy and final disappearance of the whole mass. The elaborate measures necessary to prevent excessive bleeding in all operations render them less to be desired in these conditions.

To conclude: it may be stated that in the present condition of the science the radiotherapeutic method may be used on some few cases in preference to all others; in some cases where the operative method, *cæteris paribus*, would be preferred, but in which on account of existing complications cannot be performed; and in nearly all cases, after operation, to minimize the possibility of recurrence.

Regarding the value of the ray in post-operative cases, for the purpose of minimizing to the fullest extent the likelihood of recurrence, it may be stated that herein lies the most satisfactory feature of the whole method. Scarcely any difference of opinion exists at all among surgeons who have made a practice of having their cases undergo a course of X-ray exposures after operation, as to the influence in preventing recurrence.

